# Urbanization, Land Use Behavior and Land Quality in Rural China: An Analysis Based on Pressure-Response-Impact Framework and SEM Approach

**DOI:** 10.3390/ijerph15122621

**Published:** 2018-11-22

**Authors:** Hongbin Liu, Yuepeng Zhou

**Affiliations:** 1 College of Land and Environment, Shenyang Agricultural University, Shenyang 110866, China; liuhongbinsy@syau.edu.cn; 2 China Centre for Land Policy Research & College of Public Administration, Nanjing Agricultural University, Nanjing 210095, China

**Keywords:** urbanization, household land-use behavior, cultivated land quality, pressure-response-impact (PRI) framework, structural equation model (SEM)

## Abstract

During the last 40 years, China has undergone rapid urbanization which has resulted in land degradation and a decrease in land. Cultivated land protection has thus become one of the most active and important aspects of land science. This study presents a pressure-response-impact (PRI) framework which may reveal the inter-correlations among households’ land-use behavior and cultivated land quality change in the process of rapid urbanization in China. The structural equation model (SEM) has been applied using a household survey dataset collected in 2015 in Sujiatun district, Shenyang city, Liaoning province. The results show that: (1) there is a complex causal relationship between the latent variables urbanization, household land-use behavior and cultivated land quality (i.e., urbanization → land-use behavior → land quality), which supports our PRI conceptual framework; (2) the changes of external social-economic context stemming from urbanization are the major cause of land-use behavior variance; (3) land quality is mostly affected by farmers’ land-use behavior including land-use pattern, land-use degree and land-input intensity, in particular the growing of cash crops (GCC, associated with land use pattern) and capital input per unit of farmland (LII, associated with land input intensity). These findings are of some theoretical and practical significance. Theoretically, they add to the current literature by identifying the roles of sociological factors and farmers’ land-use behavior in the process of land quality protection using a PRI framework. Practically, measures should be taken to reasonably set the prices of agricultural products, promote the development of the land rental market and increase the comparative revenue of agricultural production, so as to stimulate incentives to farming and land quality protection.

## 1. Introduction

During the last 40 years, China has undergone rapid urbanization, with the urban population increasing from 17.92% in 1978 to 57.35% in 2016 [1]. In the process of rapid urbanization, land issues, characterized by the decrease in quantity and quality, have been one of the most noticeable challenges [2]. The quantity and quality of cultivated land is closely related to national food security, sustainable agricultural production and public health [3,4]. Hence, in response to these concerns, the Chinese central government launched two principal campaigns with the aim of maintaining the quantity and quality of cultivated land across China [5]. Cultivated land protection has thus become a pressing concern.

As the basic decision-making units that could directly control land use and management, farm households play an important role in cultivated land protection [6], especially in raising the quality of cultivated land [7]. As an actor in the process of economic development, their decision on land use has been affected by the external context, e.g., economic policies relating to urbanization. In other words, economic policies relating to urbanization do not result in land quality change directly. Instead, they may affect land quality change by exerting influence on farmers’ land-use behavior.

A small amount of literature has studied the effects of urbanization and the driving forces of farmers’ land-use behavior and cultivated land quality change [7,8,9,10,11,12,13]. For example, using spatial analysis from the perspective of ecology and econometric analysis, respectively, Liang et al. and Deng et al. analyzed the impacts of urbanization on farmland changes [2,14]. They found that rapid urbanization, characterized by the changes of economic structure, social structure, employment structure and rural land institution, was one of the driving forces of farmland loss and fragmentation. By designing a conceptual framework, Kong et al. analyzed the mechanism of farmers’ land use decision-making. They concluded that farmers’ land-use behavior, including land use pattern and land quality protection, was determined by the land use target (farmers’ demand for food production, profit or both) [8]. Farmers’ land-use behavior has been widely regarded as a key influencing factor of household welfare [9,10,11]. As the human activities’ intervention on land increases, the effect of farmers’ land use behavior, especially plantation structure and land-related inputs [4], on land quality and the agro-ecological system has received much attention [7,12,13,15]. Take the study of Chen et al., for example: based on household survey and the economic statistical method, they found that farmers’ land-use behavior, including the choice of land-use type, the use of fertilizers and crop residue management, had significant impacts on the soil nutrient balances [7].

These studies have provided insights into the relationship between urbanization, farmers’ land-use behavior and cultivated land quality. However, there is room for improvement. First, rare studies have clarified the mechanism of how farmers’ land-use behavior may affect land quality in the context of urbanization. Second, to the best of our knowledge, no study has yet distinguished the direct and indirect effects of urbanization, farmers’ land-use behavior and cultivated land quality. Third, most of the extant literature employs spatial analysis or econometric methods to examine the links among the three factors. However, the inter-correlations among urbanization, farmers’ land-use behavior and cultivated land quality involve multi-variable causal effects. Those mentioned methods neglect the possibility that sociological factors may also affect household economic behavior and land quality and thus may not address the complexity sufficiently. A structural equation model (SEM) may cover this shortage.

To fill the gaps in this study, a SEM was constructed to capture the inter-links among urbanization, farmers’ land-use behavior and land quality. This was achieved by using a household survey and soil sampling. The conceptual framework of the study is summarized in the next section.

## 2. Materials and Methods

### 2.1. Conceptual Framework

**Figure 1:**
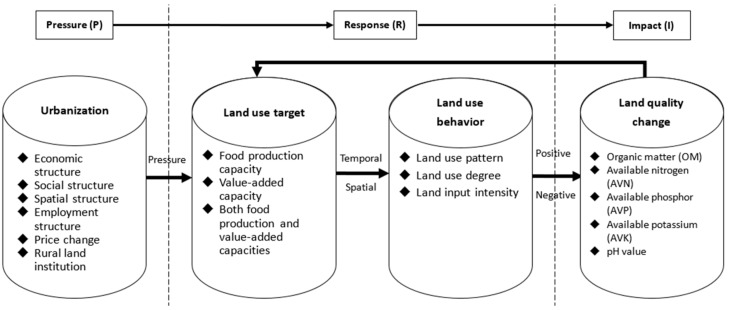
The theoretical framework of pressure-response-impact (PRI) based on households’ land-use behavior.

Originally developed by the United Nations to assess and monitor sustainability (the pressure-state-response framework), an extended ‘driver-pressure-state-impact-response’ (DPSIR) framework is a tool for detailed analysis that integrates economic, social and natural systems into a systemic approach. The DPSIR method presents mechanisms for analyzing and depicting environmental problems in an integrative way [16,17,18,19]. Extracting from the DPSIR framework, we build a ‘pressure-response-impact’ (PRI) framework (see Figure 1) which could be applied to analyze an individual household’s behavior and is more suitable to our research question. Specifically, following the pathway of ‘pressure from urbanization—household’s response on land-use based on land-use target—impact on land quality change’, we aimed to uncover the causal links between urbanization, land-use behavior and land quality change.

#### 2.1.1. Land-Use Behavior in the Process of Urbanization: The Influencing Factors and Mechanism

Accelerated development of urbanization, either expansion of large cities and regions or expansion of small towns and rural villages, has resulted in a series of changes in economic structure, social structure, spatial structure, employment structure, price mechanism, land institution, etc. Under the pressure of an external socio-economic environment, as rational-economic decision-makers, farm households would adjust their land-use targets, i.e., for food production capacity, value-added capacity, or both food production and value-added capacities, in accordance with the changes of external context and owned essential production factors (including land, labor, capital and technology) [8]. Food production was mainly to meet the demand of rural households (food production capacity). When the food demand was met, households would reduce investment in grain production and instead plant cash crops to improve the profitability of land use (value-added capacity). In particular, the adjustment of socio-economic structure stemming from urbanization would: (a) increase the employment opportunities in peri-urban areas and accelerate the labor migration from rural to urban areas [20]; (b) increase the frequency of land expropriation and lead to cultivated land loss and land tenure insecurity [14]; (c) raise the prices of means of production and agricultural products [21]. All these may stimulate or hinder famers’ land-use behavior, including land-use pattern, land-use degree and land input intensity. In addition, policies such as agricultural subsidies and land tenure reforms may accelerate the land transfer, facilitate scale-farming and encourage investment in land [22,23].

#### 2.1.2. The Influencing Mechanism of Land-Use Behavior on Land Quality

Land quality can be defined in different ways, but the soil nutrient changes and the nutrient balances are taken as key indicators of land quality [7]. Land-use behavior may greatly affect the direction and extent of soil nutrient changes and balances. Farmers’ land-use behavior could be represented by their decisions on plantation structure, land-use degree and land input intensity. Different plantation structures, i.e., the plantation of grain or cash crops, require different nutrient budgets and balances including nitrogen (N), phosphorus (P), potassium (K), etc. Land-use degree refers to the degree of interference with the land and the agro-ecosystem. Land-use degree can be represented by the multiple cropping index (MCI), which is defined as the total sowing area as a percentage of the total arable land for the same plot in one year [7]. Continuous and intensive cropping without restoration of soil fertility would deplete the nutrients of most soils, and nutrient balances are negative, indicating land degradation [7]. Land input intensity, e.g., the input of organic or chemical fertilizers, has a more direct impact on land quality. Rational investment in land could supply more nutrients essential to the growth of crops, improve the nutrient balance in the soil. Whereas, improper inputs could damage land quality, such as soil hardening and salinization. Experienced farmers could perceive the change of nutrients in the soil and thus adjust their land-use target, pattern, degree and input intensity based on it. 

### 2.2. Materials and Methods

#### 2.2.1. Study Sites and Data Collection

**Figure 2:**
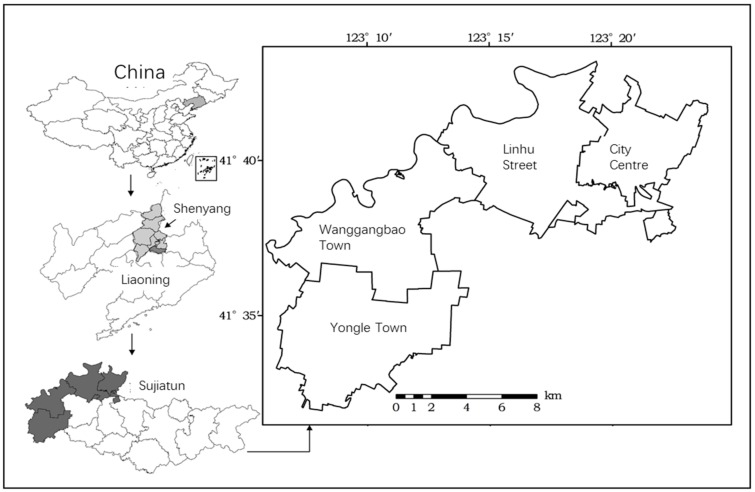
Geographical location of study sites.

This study was conducted in 2015 in Sujiatun district, Shenyang city, Liaoning province. Based on the distance to the city centre of Sujiatun, as shown in Figure 2, and the soil sampling, Linhu street (suburb), Wanggangbao town (middle) and Yongle town (outer suburb) have also been selected for the following reasons: First, the study sites are located in the middle area of Liaohe Plain and Shenyang economic zone, where the conflict between cultivated land quality protection and socio-economic development is prominent. Second, the study sites are the main supply locations of vegetables and fruits for Shenyang city. Thus, cultivated land quality protection is important for farm product outputs and food security. Third, the study sites have been strongly affected by industrialization and urbanization [13]. Farmers in different areas are influenced by different socio-economic contexts, which would lead to different land-use behaviors and cultivated land quality status. As a result, the selection of study sites is representative.

After the selection of study sites, the field survey was conducted in July–August. Based on the town size, a number of villages and households were selected randomly in each town. Data were collected via a questionnaire which covered household characteristics, land-use behavior, and soil sampling. 

Soil samples were collected during May–June (after soil thawing and before fertilization). At each plot, 15–20 surface soil samples at 0–20 cm depth were collected using the chessboard method. The soil samples were well blended, equally divided and put into four 20 × 15 cm plastic bags (1 kg each), which were labeled showing soil sample number, plot name, GPS location, the farmers’ names and addresses, and the date of collection.

A total of 240 questionnaires were collected. By excluding two invalid questionnaires, the sample was reduced to 238.

#### 2.2.2. Model Specification and Variable Selection

Following our pressure-response-impact (PRI) conceptual framework, the empirical data is analyzed using SEM. Structural equation modeling is often used in a causal or path analysis framework which allows a simultaneous estimation of several equations of independent and dependent variables, thus allowing a multi-layered model to be assessed. Structural equation modeling is a technique that can handle a large number of endogenous and exogenous observable variables simultaneously [24,25]. A structural equation model consists of: (a) the measurement models, which link the observable variables to the latent variables; and (b) the structural part, which links the latent variables to each other using systems of simultaneous equations [26]. A latent variable refers to a phenomenon that is supposed to exist but cannot be directly observed. It is given empirical meaning (measured) by means of correspondence statements that relate it to a set of observable variables (indicators) [27]. In this study, four latent variables were selected (as shown in Table 1):

(1) Urbanization, which could be captured by eight observable variables, including distance from the sample village to town centre (VCD), frequency of land adjustment (LAN), number of off-farm employment members (NFN), average price of agricultural products (APP, yuan/kg), average price of agricultural means of production (MPP, measured by average price of chemical fertilizers, yuan/kg), number of plots (LN), agricultural subsidy received in total (AST, yuan), and frequency of participation in technology training (TTN) [2,10,14,28].
(2) Internal factors, which contain six observable variables, i.e., age of household head (AGE), education level of household head (EDU), years engaged in agricultural production (YEAR), number of agricultural laborers in the family (ALN), household’s annual income (HIT), and farmland area (LRN) [28,29].
(3) Land-use behavior, which includes: grow cash crop (GCC), MCI and capital input per unit of farmland (LII) [30].
(4) Land quality, which is indicated by five observable variables, i.e., organic matter (OM), available nitrogen (AVN), available phosphorus (AVP), available potassium (AVK), and pH value [28,31].

Equations (1) and (2) present the measurement models for the endogenous and exogenous variables, respectively [27].

y = Λ_y_ η + ε (1)

x = Λ_x_ ξ + δ (2)

where y is a vector of endogenous observable variables, x is a vector of exogenous observable variables, η is a vector of latent endogenous variables, and ξ is a vector of latent exogenous variables. Λ_y_ and Λ_x_ are matrices of coefficients (or loadings). Finally, ε and δ are vectors of measurement errors of y and x, respectively.

The structural model is represented by Equation (3):

η = B η + Γ ξ + ζ (3)

where η and ξ are defined in (1) and (2), B is a matrix with β_ij_ representing the effect of the *j*th endogenous latent variable on *i*th endogenous latent variable, Γ is a matrix with γ_ij_ representing the effect of the *j*th exogenous latent variable on *i*th endogenous latent variable, ζ is a vector of disturbances.

**Figure 3:**
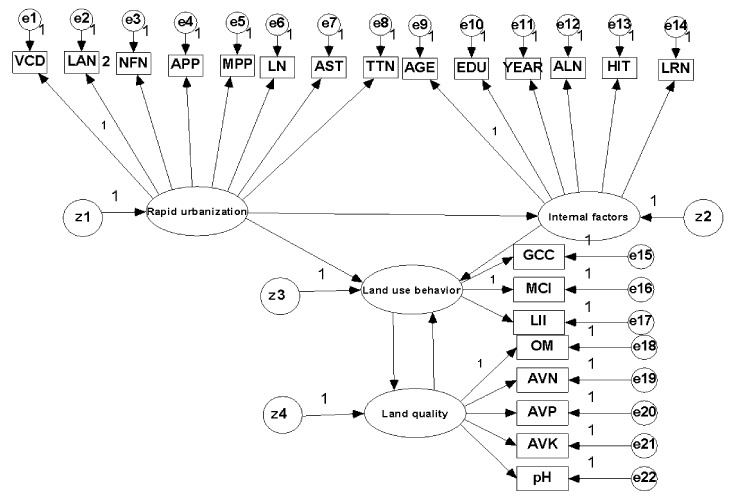
The structural equation model (SEM) of the land-use behavior to land quality in the process of urbanization.

Because of the ability of SEM to specify complex underlying relationships, graphical representation, such as Figure 3, has become the standard means for presenting information about SEM.

## 3. Results

**Figure 4:**
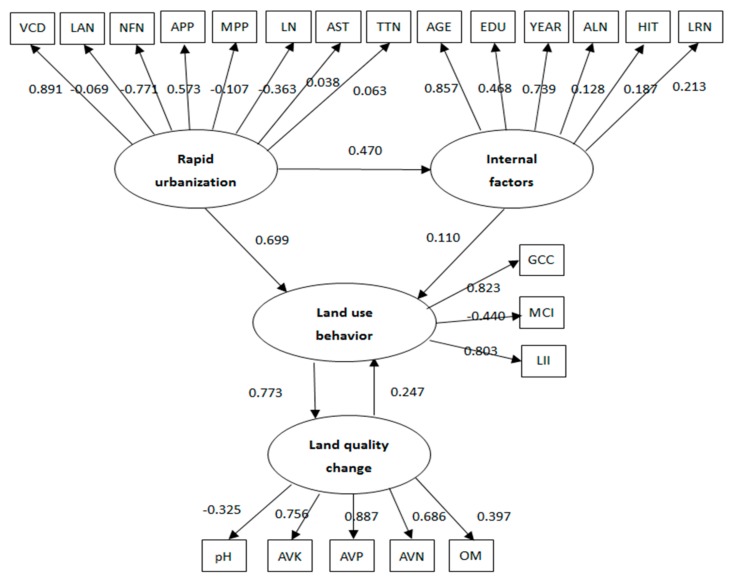
The structural equation diagram for urbanization-land use behavior-land quality analysis.

The SEMs in Figure 3 were estimated using AMOS 17.0. Table 2 reports the estimated results for both the measurement model and structural model. The χ^2^ coefficient for the SEM is 338.131 (df = 204, *p* = 0.000, PMSEA = 0.043, NFI = 0.903, CFI = 0.919), indicating that the SEM fits the data well. The graphical representation of Table 2 is shown in Figure 4. Table 3 reports the standardized direct, indirect and total effects between latent variables.

### 3.1. The Influencing Factors of Land-Use Behavior

With regards to the influencing factors of farmers’ land-use behavior, the coefficients (shown in Table 2) for urbanization and internal factors have better explaining power, which also proves that the PRI framework is acceptable. Urbanization and internal factors both have positive impacts on land-use behavior, significant at the level of 1% and 5%, respectively. The direct effects (represented by the standardized coefficient) of urbanization and internal factors on land-use behavior are 0.699 and 0.11, respectively. This indicates that, with other conditions unchanged, farmers’ land-use behavior would rise by 0.699 and 0.11 when urbanization and internal factors increase by one unit. As can be seen in Table 3, the indirect and total effects for pathway ‘urbanization → internal factors → land-use behavior’ are 0.299 and 0.928. Consequently, compared to internal factors, the changes of external social-economic context stemming from urbanization are the major determinants of land-use behavior. Moreover, urbanization also has a positive impact on a household’s internal factors. Rapid urbanization facilitates the improvement of household endowment.

For the eight observable variables representing urbanization, VCD, APP, AST and TTN have significant and positive impacts on land-use behavior. With VCD, APP, AST, and TTN increased by one unit, land-use behavior would increase by 0.891, 0.573, 0.038 and 0.063, respectively. In other words, increasing the distance from the town centre, raising the price of agricultural products, increasing agricultural subsidy and increasing the frequency of technology training would make farmers’ land-use behavior more sustainable. Among these, regional location and price of agricultural products are the main factors. 

Frequency of land adjustment, NFN, MPP and LN have significant and negative effects on land-use behavior, valued at −0.069, −0.771, −0.107 and −0.363, respectively. Therefore, land tenure insecurity, increasing number of off-farm labor force, rising price of farm-related inputs, and land fragmentation would all impede the sustainability of land-use behavior. Among them, the number of off-farm labor force and land fragmentation are the main factors. 

The internal factors, including AGE, EDU, YEAR, ALN, HIT, and LRN, all have significant and positive impacts on land-use behavior. The standardized coefficients are 0.857, 0.468, 0.739, 0.128, 0.187 and 0.213, respectively. Age, education and farming experience of household head are the main factors, which indicate that the ability of the decision maker is of more importance than other internal factors.

### 3.2. The Impact of Land-Use Behavior on Land Quality

With regards to the influencing factors of land quality, the results shown in Table 2 indicate that farmers’ land-use behavior has a significant and positive effect, with the standardized coefficient valued at 0.773. In turn, land quality also imposes an effect on farmers’ land-use behavior, with the standardized coefficient valued at 0.247. In other words, while land quality could be largely improved by guiding farmers’ land-use behavior, it can also act back on famers’ land-use behavior. Moreover, as is shown in Table 3, the indirect and total effects of ‘urbanization→land-use behavior→land quality’ are both 0.717, implying that, with an urbanization increase of one unit, land quality would rise by 0.717.

For the three observable variables representing land-use behavior, the standardized coefficients for GCC and LII are 0.823 and 0.803, respectively. The positive effects of land-use behavior imply that farmland on which cash crop was grown and more capital input per unit of land was received is associated with higher soil quality. This is because, in the study sites, the planting of cash crops (vegetables, fruits) requires the application of base fertilizer (manure) which is beneficial to soil. The higher capital input means more chemical fertilizer inputs, which is also a good supplement to soil nutrient balances when it is applied appropriately.

The multiple cropping index, however, has a negative effect on land quality, as expected. Land that grows multiple crops indicates more intensified use. The more crops grown, the more nutrients, such as N, P and K, are absorbed, and this leads to land degradation. In addition, the diversity of planted crops (high MCI) may result in land fragmentation, which would affect fertilization negatively [30].

For the five observable variables representing land quality, AVN, AVP and AVK are mostly affected by farmers’ land-use behavior, with the standardized coefficient valued at 0.686, 0.887 and 0.756, respectively. The finding provides strong support in our conceptual framework that land-use behavior, especially inputs of N, P and K, may have the strongest and most direct effect on land quality. The influence of OM is relatively weak, though positive as well. This can be explained by the fact that, in our survey area, farmers paid little attention to input of organic fertilizer (such as manure), which leads to the excessive use of chemical fertilizers. As a result, the soil is acidized and the influence of pH value is negative.

## 4. Conclusions and Policy Implications

The impact of farmers’ land-use behavior on land quality in the process of urbanization has received much attention in various research fields. The studies on the relationship between land-use behavior and land quality have made considerable progress. However, their causal links and interaction mechanism in the context of urbanization are still unclear. To fill this gap, we built a pressure-response-impact (PRI) framework and employed SEMs to uncover how urbanization, land-use behavior and land quality interact. 

The main findings of this study are as follows. First, it is proved that urbanization, land-use behavior and land quality indeed interact with each other. Second, the changes of external socio-economic context stemming from urbanization are the major determinant of land-use behavior. Specifically, VCD and APP have the strongest positive impacts on land-use behavior, while both the NFN and the LN have the strongest negative impacts. Third, land quality is mostly affected by farmers’ land-use behavior, in particular GCC and LII.

These findings are of some theoretical and practical significance. Theoretically, they add to the current literature by identifying the roles of sociological factors and farmers’ land-use behavior in the process of land quality protection using a PRI framework. Practically, some policy implications can be made based on our findings. First, reasonably set the prices of agricultural products and increase the comparative revenue of agricultural production. Low prices and low comparative revenue would greatly dampen incentives to farming and thus adversely affect land quality protection. Second, the development of land rental market and moderate scale farming may be an effective way of reducing land fragmentation and thus improving land quality.

It should be noted that land quality change is a dynamic and complex process. Soil nutrients such as N, P, K, and pH value, could not fully represent land quality. Land quality indicators related to crop productivity can also include crop yields, oxygen availability, salinity and sodicity, etc. [4]. Moreover, a cross-sectional dataset could not capture the trend of land quality changes, the actual nutrient use efficiencies, nutrient cycles, or biophysical soil health in a long period. It is important for further research to introduce more indicators of land quality, set up fixed observation points in rural China and collect panel data, so as to trace the changes of land quality and examine the complex interaction among urbanization, land-use behavior and land quality more rigorously from both temporal and spatial dimensions.

## Figures and Tables

**Table 1:** Definition and descriptive statistics of latent and observable variables.

Latent Variables	Acronym	Code	Definition of Observable Variables	Type	Mean	S.D.	Min.	Max.
**Urbanization**	VCD	e1	Distance from the sample village to town centre (km)	continuous	13.94	5.83	5.70	21.70
	LAN	e2	Frequency of land adjustment	discrete	1.1	1.8	0.0	10.0
	NFN	e3	Number of off-farm employment members	discrete	2	1	1	5
	APP	e4	Average price of agricultural products such as rice, corn, and wheat (yuan/kg)	continuous	2.52	2.22	0.20	12.40
	MPP	e5	Average price of agricultural means of production such as fertilizers, manure and pesticides (yuan/kg)	continuous	8.00	5.18	0.60	23.60
	LN	e6	Number of plots	discrete	2	1	1	5
	AST	e7	Agricultural subsidy received in total in 2014 (yuan)	continuous	652	659	55	6860
	TTN	e8	Frequency of participation in technology training	discrete	3	10	0	99
**Internal factors**	AGE	e9	Age of respondent farmer (years)	continuous	53	11	25	88
	EDU	e10	Education of respondent farmer (years)	discrete	8	2	2	13
	YEAR	e11	Length of years engaged in agricultural production (years)	discrete	28	15	5	70
	ALN	e12	Number of agricultural laborers in the family	discrete	2	1	1	6
	HIT	e13	Household annual income (yuan)	continuous	51,896	71,061	786	771,960
	LRN	e14	Farmland area (hectare)	continuous	0.88	0.83	0.07	8.00
**Land-use behavior**	GCC	e15	Grow cash crop, 1 = yes; 0 = no	dummy	0.6	0.4	0	1
	MCI	e16	Multiple crop index = total sowing area/total land area	continuous	1.3	0.5	1	3
	LII	e17	Capital input per unit of farmland (yuan/hectare)	continuous	16,215	16,485	1785	67,680
**Land quality**	pH	e18	pH value	continuous	5.8	0.6	4.8	8.4
	AVK	e19	Available potassium (mg/kg)	continuous	200.5	148.5	82.3	833.7
	AVP	e20	Available phosphorus (mg/kg)	continuous	167.5	169.5	7.1	800.8
	AVN	e21	Available nitrogen (mg/kg)	continuous	138.0	37.9	77.0	314.0
	OM	e22	Organic matter (g/kg)	continuous	26.8	6.5	15.3	51.7

Sources: computed based on household survey data and laboratory analysis of the soil samples.

**Table 2:** Model path coefficient estimation results.

Relation			Coef.	S.E.	C.R.	P	Std. Coef.
***Structural model:***							
Internal factors	←	Urbanization	0.651	−0.147	−5.806	***	0.47
Land-use behavior	←	Urbanization	0.855	0.008	6.507	***	0.699
Land-use behavior	←	Internal factors	0.005	0.003	1.977	*	0.11
Land quality	←	Land use behavior	4.888	0.870	5.618	***	0.773
Land-use behavior	←	Land quality	0.039	0.020	1.994	*	0.247
***Measurement model:***							
VCD	←	Urbanization	1	—	—	—	0.891
LAN	←	Urbanization	−0.024	0.012	−1.996	*	−0.069
NFN	←	Urbanization	−0.227	0.091	−2.482	***	−0.771
APP	←	Urbanization	0.122	0.014	8.774	***	0.573
MPP	←	Urbanization	−0.053	0.021	−2.545	**	−0.107
LN	←	Urbanization	−0.071	0.013	−5.362	***	−0.363
AST	←	Urbanization	4.854	1.903	2.551	**	0.038
TTN	←	Urbanization	0.121	0.042	2.913	**	0.063
AGE	←	Internal factors	1	—	—	—	0.857
EDU	←	Internal factors	0.102	0.016	6.464	***	0.468
YEAR	←	Internal factors	1.203	0.131	9.165	***	0.739
ALN	←	Internal factors	0.169	0.085	1.99	*	0.128
HIT	←	Internal factors	0.021	0.008	2.614	***	0.187
LRN	←	Internal factors	1611.88	541.626	2.976	***	0.213
GCC	←	Land-use behavior	1	—	—	—	0.823
MCI	←	Land-use behavior	−0.498	0.074	−6.774	***	−0.44
LII	←	Land-use behavior	2155.236	153.256	14.063	***	0.803
OM	←	Land quality	1	—	—	—	0.397
AVN	←	Land quality	10.048	1.772	5.669	***	0.686
AVP	←	Land quality	58.055	9.618	6.036	***	0.887
AVK	←	Land quality	43.352	7.428	5.836	***	0.756
pH	←	Land quality	−0.072	0.019	−3.889	***	−0.325
χ^2^			338.131				
df			204				
RMSEA			0.043				
CFI			0.919				
NFI			0.903				

Note: *** 1%, ** 5%, and * 10%.

**Table 3:** Standardized direct, indirect and total effects between latent variables.

Pathways	Std. Coef.		
	Direct Effect	Indirect Effect	Total Effect
Urbanization → Internal factors	0.47	—	0.47
Urbanization → Land-use behavior	0.699	0.229	0.928
Urbanization → Land quality	—	0.717	0.717
Internal factors → Land-use behavior	0.110	—	0.110
Internal factors → Land quality	—	0.105	0.105
Land-use behavior → Land quality	0.773	—	0.773
Land quality → Land-use behavior	0.247	—	0.247
